# Meloxicam in the Environment: Pathways of Entry, Ecotoxicity, and Removal Strategies

**DOI:** 10.3390/toxics14070631

**Published:** 2026-07-20

**Authors:** Semyon M. Tyan, Irina B. Ivshina

**Affiliations:** 1 Institute of Ecology and Genetics of Microorganisms of the Ural Branch of the Russian Academy of Sciences, Perm Federal Research Center of the Ural Branch of the Russian Academy of Sciences, 13 Golev Str., 614081 Perm, Russia; vviolent00@mail.ru; 2 Department of Microbiology and Immunology, Perm State National Research University, 15 Bukirev Str., 614990 Perm, Russia

**Keywords:** meloxicam, pharmaceutical pollution, non-steroidal anti-inflammatory drugs (NSAIDs), environmental effects, bioremediation

## Abstract

Meloxicam, a selective cyclooxygenase-2 inhibitor, is commonly used as non-steroidal anti-inflammatory drug in both human and veterinary medicine. Owing to its heterocyclic structure, toxicity to invertebrates, vertebrates, and microorganisms, and potential to biomagnify through the food webs, meloxicam persists in the environment and poses a risk to ecological health. There is still a paucity of information on meloxicam as a pharmaceutical contaminant. It is not included in priority lists, which means its environmental risk is often underestimated. This paper provides an integrative overview of the currently available literature (2009–2026) on the environmental occurrences, ecotoxicity, transport pathways, and removal strategies of meloxicam. The distribution of meloxicam has been most extensively studied in aquatic environments, whereas information on soils, plants, and terrestrial fauna remains limited. Bioaugmentation is the most promising strategy for reducing ecotoxicological risk, although its effectiveness against meloxicam has so far been confirmed only in a few model systems. The evidence presented here underscores the importance of strict regulation of meloxicam use and routine environmental monitoring. We hope this review will be useful to ecologists, microbiologists, and regulatory agencies developing strategies to mitigate pharmaceutical contamination.

## 1. Introduction

Pharmaceutical pollution is recognised as one of the major global environmental challenges, contributing to antimicrobial resistance and posing risks to public health [1,2,3,4]. The challenge is exacerbated by pharmaceutical consumption (an increase of 6.5% annually, based on the 2019 data) [5], demographic ageing of the population [6], an increasing share of chronic diseases [7,8,9], and intensive use of antimicrobial drugs in human medicine and animal husbandry (a projected increase of 67% by 2030) [10]. According to the National Health Service, these factors account for 20–25% of the healthcare carbon footprint, thereby posing a serious threat to biodiversity and human health [11].

Among other NSAIDs, such as diclofenac, ibuprofen, naproxen, and ketoprofen, diclofenac remains the most widely used worldwide. This is also reflected in production patterns. In a multi-country sales analysis, diclofenac was identified as the best-selling NSAID, is widely included in national essential medicines lists, and is frequently detected in environmental monitoring programmes [12,13]. However, robust global data on the production volumes of active pharmaceutical ingredients for all five compounds remain limited.

**Figure 1:**
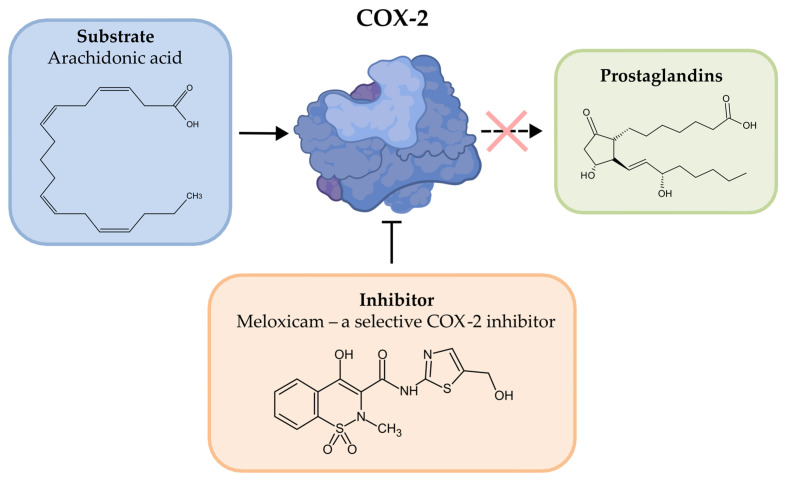
Meloxicam mechanism of action.

Among pharmaceutical active compounds, NSAIDs are of particular concern due to their persistence, ability to bioaccumulate in trophic webs, and capacity to disrupt key physiological processes in living organisms [14,15,16]. Meloxicam is a polycyclic thiazole derivative and a selective inhibitor of cyclooxygenase-2 (COX-2) (Figure 1).

Meloxicam is used to treat musculoskeletal injuries, osteoarthritis, rheumatoid arthritis, ankylosing spondylitis, and postoperative pain [17,18] (Table 1). Despite its widespread use in both human and veterinary medicine, meloxicam has been studied less extensively from an ecological perspective [19] than better-characterised drugs such as paracetamol [20], diclofenac [21], ibuprofen [22], and naproxen [23,24].

The substantial production and consumption of meloxicam (market volume is 7.97 billion USD, 2026) increase the likelihood of its release into wastewater and agricultural runoff [34]. However, its environmental priority should be determined not only by sales volumes but also by its fate, toxicity, and occurrence in environmental matrices. At present, meloxicam is not included in the list of priority pollutants, and information on its persistence, environmental distribution, transformation pathways, and ecotoxicological profile is still limited [15,35].

This review has an integrative format, because the field is characterised by substantial heterogeneity. In particular, the use of diverse experimental approaches, multiple model systems, and inconsistent data presentation makes it difficult to apply a strictly systematic protocol with uniform eligibility criteria. Accordingly, a narrative approach was used to consolidate the available evidence, outline the principal research trends, and highlight unresolved issues. An analysis of its detection and a summary of ecotoxicological data were conducted, with particular attention to bioaugmentation as a promising and sustainable strategy for detoxifying micropollutants in aquatic and terrestrial ecosystems. Addressing this significant gap will improve the accuracy of environmental risk assessments and support the development of evidence-based measures to reduce negative impacts on biodiversity and human health.

The literature search was conducted using scientific databases, including Google Scholar, Scopus, PubMed and Web of Science. The primary search focused on publications from 2009 to 2026 to capture the most recent information. Earlier studies were also included when they provided background information.

## 2. Physicochemical Properties of Meloxicam

Meloxicam is classified as a compound with high permeability but extremely low aqueous solubility [36]. This limits its bioavailability [37] and contributes to its environmental persistence [38]. The literature provides a detailed account of its acid–base behaviour, solubility, lipophilicity, and the influence of crystal form and various carriers on its physicochemical behaviour (Table 2).

Meloxicam is a weakly acidic, lipophilic compound with pH-dependent solubility and polymorphism. It is a diprotic weak acid with two pKa values: approximately 4.2, corresponding to dissociation of the enolic hydroxyl group, and approximately 1.1, corresponding to protonation of the thiazole nitrogen. These ionization constants, its pH-dependent solubility, ionisation state and lipophilicity are known [36]. At least five crystalline forms are known [42]. The key differences between the five crystalline forms of meloxicam are related to the packing of molecules in the crystal, the degree of hydration, and consequently, stability, solubility, melting point, and pharmaceutical suitability [43,44]. The structures have been detailed only for Form I and Form IV (monohydrate), while the other forms are considered anhydrous or associated with dehydration transitions [45]. In commercial practice, it is precisely Form I [45,46] that is used, which, despite its lower solubility, is considered the only pharmaceutically acceptable form due to its high stability [44].

From a pharmacokinetic standpoint, meloxicam belongs to BCS Class II [41], meaning it possesses high permeability but low solubility. This is associated with a relatively slow onset of maximum plasma concentration, typically 4–6 h following administration [47,48,49]. Thus, low solubility and moderate lipophilicity of meloxicam are likely to promote its distribution between water, suspended particles, and biota, as well as sorption in sediments and entry into food webs.

## 3. Environmental Sources of Meloxicam Entry

### 3.1. Factors Contributing to the Penetration of Meloxicam into Aquatic Ecosystems

**Figure 2:**
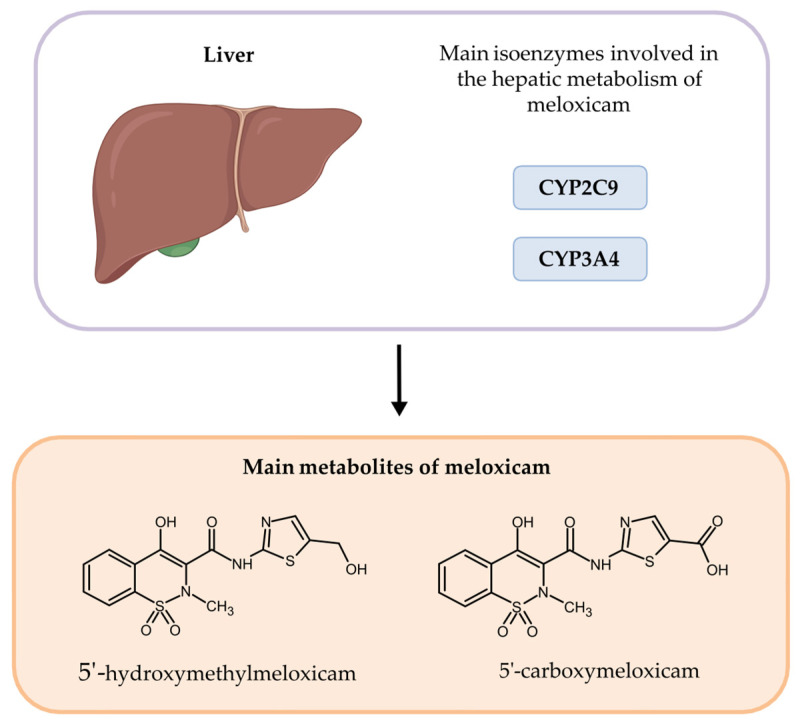
Meloxicam metabolism in the human body.

Meloxicam has been widely used in clinical practice for pain relief and anti-inflammatory therapy since 1995 [50,51]. Its pharmacological action (trade name Mobic^®^) is based on the selective inhibition of COX-2, which is induced at sites of tissue damage and responsible for prostaglandin synthesis—key mediators of inflammation [17,52]. Unlike paracetamol and opioids, which lack anti-inflammatory effects, meloxicam combines analgesic and anti-inflammatory actions, thereby accounting for its widespread use in alleviating pain, swelling, and stiffness [53]. The drug is metabolised in the liver to form 5′-carboxymeloxicam and 5′-hydroxymethylmeloxicam and is excreted principally via the intestines and kidneys as these metabolites [54] (Figure 2).

Meloxicam is produced worldwide, across regions of Asia, Europe, and the post-Soviet space. The scale of this process is substantial: annually, numerous tonnes of active substance are synthesised [34]. This makes it possible to produce millions of solid dosage forms and injection solutions, thereby satisfying global demand for meloxicam as an accessible and effective treatment for inflammatory diseases in both human and veterinary medicine [17,55,56]. Its high therapeutic efficacy and favourable safety profile (the drug was developed as a less toxic alternative to diclofenac sodium) underpin its popularity [27,57,58]. However, such intense industrial activity inevitably generates significant quantities of industrial waste, thereby increasing anthropogenic load on natural ecosystems [59]. Increased life expectancy is guaranteed by the mass manufacture and distribution of pharmaceuticals, but it also increases pharmaceutical emissions into the environment, which has a negative impact on human health [60,61,62].

Meloxicam enters aquatic ecosystems through several pathways. Its large-scale production inevitably generates substantial industrial waste, and up to 5% of the drug is excreted unchanged [18]. Furthermore, pharmaceuticals, such as meloxicam, are frequently flushed down the toilet or dumped in municipal solid waste dumps [63,64]. Because of the limitations of conventional treatment techniques, the efficacy of current wastewater treatment facilities with respect to micropollutants is still low [65]. The direct release of waste from animal farms is particularly relevant, as meloxicam is frequently used as an anti-inflammatory agent in pigs, cattle, rabbits, and horses [66,67]. This increases bioaccumulation and long-term dangers for aquatic biota since a certain amount of the drug and its metabolites infiltrate surface and groundwater with a likely retention of biological activity.

Meloxicam is mostly absent from international studies of pharmaceutical pollution. This NSAID is not covered by the systems for eco-pharmacovigilance [68] and efforts for evaluating the environmental risk of medicines [69] that are being put into place in a number of nations. There are also no established maximum permissible residual levels for meloxicam [70]. The World Health Organization and the United Nations Environment Programme are crucial in categorising substances that need to be regulated and creating international standards (such as the Stockholm Convention on Persistent Organic Pollutants) [71]. The U.S. Environmental Protection Agency, the European Chemicals Agency, and the Food and Drug Administration prioritise chemicals based on parameters of persistence, bioaccumulation, and toxicity. We assume that determining the environmental distribution of meloxicam is challenging. The heterogeneity of monitoring data and the lack of comparable emission assessments complicate the inclusion of the NSAID in the list of priority pollutants. However, despite the limited available literature, the presented information allows us to reasonably suggest that meloxicam may pose a potential threat to the biosphere.

Because the population is ageing and chronic conditions are becoming more common, a potential increase in the prescription of NSAIDs (particularly meloxicam) is expected [72]. In the long term, such a situation may increase the burden on the environment. The amount and kinds of medicines that enter aquatic environments will rise as the world’s population expands, particularly in metropolitan areas [73], unless substantial adjustments are made to manufacturing methods, consumption habits, and preventative measures. However, robust quantitative forecasts require the integration of drug consumption, changes in prescribing practices, and monitoring data.

Therefore, the primary causes of meloxicam’s environmental penetration are the flaws in wastewater treatment systems, the agricultural dumping of manure containing drug residues, and the absence of international designation of meloxicam as a priority contaminant.

The main presumed pathways for the entry of meloxicam into the environment are mainly incomplete excretion from the body, use in veterinary medicine, disposal of unused medications, and insufficient efficiency of wastewater treatment facilities.

### 3.2. Methods for Detecting Meloxicam in the Environment

The detection of pharmaceutical pollutants in environmental samples has become possible due to the development of sensitive analytical methods and modern sample techniques, including high-performance liquid chromatography (HPLC-MS), gas chromatography–tandem mass spectrometry (GC-MS/MS), high-resolution mass spectrometry (HRMS), etc. [16]. Ultra-pressure liquid chromatography/quadrupole time-of-flight/mass spectrometry (UHPLC-QTOF-MS) is used to monitor meloxicam and its transformation products in aquatic environments. This allows for the detection of meloxicam at trace levels in river water [74].

For wastewater treatment and bioremediation studies, HPLC-MS is used to monitor decomposition kinetics and identify by-products [75]. In several studies, solid-phase extraction (SPE) combined with LC-MS/MS has been used to improve analytical sensitivity [74]. SPE remains the most commonly used sample preparation step for environmental matrices because it enables analyte preconcentration and reduces matrix complexity. For example, meloxicam was extracted from 1000 mL wastewater samples by SPE, followed by evaporation and reconstitution, yielding an estimated enrichment factor of approximately 10,000 [76].

SPE and the use of large sample volumes offer similar advantages for the analysis of related xenobiotics. Preconcentration by SPE from large volumes of water can reduce detection limits to the µg/L range and below [77,78].

As a more accessible option for routine monitoring, high-performance liquid chromatography with ultraviolet detection (HPLC-UV) is often employed. Although it is less selective than LC-MS/MS in complex environmental matrices, it remains simpler and more economical to implement. For meloxicam, these methods typically use reversed-phase C_18_ columns with detection at 360–364 nm [79,80]. Micellar electrokinetic chromatography (MEKC) combined with off-line SPE also allows achieving sensitivity at the ng/L level in industrial wastewater with good separation in less than 10 min [76]. Spectroscopic methods are primarily useful as rapid screening tools: attenuated total reflectance Fourier-transform infrared spectroscopy (ATR-FTIR) combined with principal component analysis (PCA), partial least squares regression (PLSR), and soft independent modelling of class analogy (SIMCA) allowed for the detection of meloxicam in milk down to 1 µg/kg [81]. High-performance thin-layer chromatography (HPTLC) and electrochemical approaches are considered more cost-effective alternatives, especially for routine and field studies [82]. Finally, high-resolution mass spectrometry (HRMS) and non-targeted screening are gaining increasing importance in environmental analysis, as they are particularly effective for identifying unknown transformation products and trace analytes of exact mass [83].

Thus, the optimal strategy for detecting meloxicam in environmental samples is a combination of SPE and LC-MS/MS for confirmatory analysis, while HPLC-UV, MEKC, ATR-FTIR chemometrics, and electrochemical methods should be considered as more accessible options.

### 3.3. Detection of Meloxicam in the Environment

It is important to remember that a number of factors affect the detection of pharmaceutical active compounds and their concentration in the environment. These include the source of wastewater, the layout of treatment plants, the climatic conditions, the processes of dilution and dispersion, and the existence of metabolites and other products like excipients [84,85].

NSAIDs are presently often and widely found in surface waters, wastewater, and agricultural soils due to their extensive usage and inadequate removal in conventional treatment facilities [19,86,87]. Persistent pharmaceutical chemicals can move from manure, different livestock waste, and irrigation water into soil and groundwater under agroecosystem settings, causing long-term pollution and possible injury to people, animals, and plants [88].

The literature on the detection of meloxicam in environmental matrices remains highly fragmented, despite its global production and use. This limits robust ecological risk assessment. Reported concentrations in water matrices include 0.2 ng/L in drinking water and 3.9 and 1.5 ng/L in incoming and processed wastewater in Catalonia (Spain) [89]. Higher concentrations have been reported in wastewater and the Danube River (Serbia)—1.8–5.0 ng/L [90], in the wastewater of Athens (Greece)—6.54–218 ng/L [91], and in Liaoning Province (China), where concentrations reached 90–800 ng/L [92]. The worldwide character of the issue is shown by the discovered geographical heterogeneity; yet, the creation of appropriate standards and risk minimisation methods is hampered by the crucial absence of monitoring data across the majority of the world.

Humans and predators, as end consumers of animal products, may be exposed to pharmaceutical active compounds that bioaccumulate in tissues and transfer through food webs.

Meloxicam is used in veterinary medicine to treat mastitis and dysmenorrhea in cattle, postpartum sepsis and mastitis-metritis-agalactia syndrome in pigs, and inflammatory musculoskeletal disorders in sheep, which leads to its introduction into agroecosystems [93,94,95]. The occurrence of residual amounts is confirmed by regulatory limits in the PRC. The maximum permissible levels of meloxicam in the liver (65 µg/kg), muscle tissue (20 µg/kg), and milk (15 µg/kg) of cattle emphasise the risk to dairy consumers and newborn calves [96].

The information on meloxicam’s prevalence in Iberian Peninsula animals is especially important. The compound has been detected in the kidneys of raptors in Catalonia at concentrations of 0.231–0.264 mg/kg and in their liver at 0.159–641 mg/kg, as well as in pig liver (0.023 mg/kg) in Valencia, Spain [67]. Particular concern arises from the detection of meloxicam in the eggs of bearded vultures (*Gypaetus barbatus*) from Andalusia, Spain [97]. Therefore, systemic bioaccumulation of meloxicam in farmed and wild animals provides a persistent pathway into food webs and may affect human health.

The development of techniques for meloxicam detection in different matrices is still an important endeavour, despite the paucity of data on its identification in natural objects [98]. Due to the concentration of research on priority pollutants, information on more widely used NSAIDs predominates in the literature that is now accessible [99,100]. However, the available evidence confirms that meloxicam is widely distributed across the biosphere and has entered ecosystems, mostly at trace levels. The geographical heterogeneity indicates an insufficiency of monitoring, rather than a localised pattern of pollution, likely underestimating the substance’s true spread. Meloxicam is detected both in wastewater and surface waters, as well as in the tissues of agricultural and wild animals, including reproductive organs, which indicates its ability to enter trophic webs.

From an ecological perspective, the most significant aspect is its capacity to persist in different matrices and move between aquatic environments, agroecosystems, and living organisms. The detection of residual amounts in the liver, kidneys, and eggs of predatory birds points to the systemic meloxicam distribution and its accumulation, which is especially critical for species at the top levels of the food web. Therefore, even at low concentrations, meloxicam should be considered a potentially important pharmaceutical active compound capable of exerting a long-term effect on the structure and functioning of ecosystems.

## 4. Ecotoxicity of Meloxicam

As was previously said, wastewater, agricultural runoff, and inappropriate disposal are the major ways that pharmaceutical substances get into the aquatic environment and remain there since treatment systems do not completely remove them. At ecologically important quantities, typically between ng/L and µg/L, this poses a long-term risk to non-target species [101]. These pollutants have been shown to have a variety of biological impacts, such as oxidative stress, histological damage, and disruption of aquatic creatures’ physiological processes. In order to establish bioremediation techniques and regulatory frameworks, it is imperative to evaluate the toxicity of all pharmaceutical contaminants [102]. The risk quotient (RQ = MEC/PNEC), calculated from the measured environmental concentration (MEC) and the predicted no-effect concentration (PNEC), is widely used in environmental assessments as a screening metric. It compares the measured concentration in water with the concentration below which adverse effects are not expected, thereby facilitating prioritisation of pharmaceutical pollutants for monitoring and management [103]. In the standard interpretation, RQ > 1 indicates a potential ecological risk and serves as a trigger for further assessment, monitoring, and management measures [104].

The calculation of RQ for meloxicam is methodologically acceptable, provided reliable MECs and a justified PNEC value [103,105,106]. However, the RQ serves as a screening indicator of threshold exceedance [107]. For meloxicam, RQ is calculated according to the standard approach to pharmaceutical contaminants. The monitored MEC (surface water, effluents, etc.) is divided by a PNEC derived from ecotoxicological endpoints and an appropriate assessment factor [106]. Limitations of the RQ approach include (a) a lack of ecotoxicological data; (b) reliance on a single-point MECs that do not account for seasonality and peak concentrations; and (c) the occurrence of pharmaceuticals in environmental mixtures, for which mixture-based approaches are preferred when assessing cumulative effects.

### 4.1. Acute Toxicity of Meloxicam

Acute toxicity may be objectively measured using median lethal concentration (LC_50_) or median effective concentration (EC_50_) indicators. Acute toxicity is defined as an unfavourable impact that arises from a single exposure to a high dosage or short-term exposure to a chemical and manifests within a few hours or days.

There is scant information on the acute toxicity of meloxicam, most of which relates to aquatic creatures. Acute exposure to the common carp *Cyprinus carpio* (LC_50_ for 24 h = 10.05 mg/L) causes fast physiological disruptions, including damage to the liver and gills and elevated levels of erythrocytes, haematocrit, haemoglobin, and malondialdehyde (a sign of lipid peroxidation) [102].

Meloxicam (100 mg/mL) treatment in the roundworm *Caenorhabditis elegans* results in abnormalities in spontaneous movement, suggesting harm to neurones and the neural network. Neurotoxicity is specifically indicated by a reduction in body surface area [108].

Review studies on the ecotoxicity of NSAIDs in freshwater invertebrates reveal that while chronic sublethal effects (on growth, reproduction, and metabolic indicators) occur at values of ng–µg/L, acute fatality is only shown at unrealistically high doses [109]. Although meloxicam is not included in these evaluations, the pattern shows that chronic endpoints (behaviour, development, and reproduction) show hazardous effects at much lower doses than LC_50_ ones, indicating their higher sensitivity (acute-to-chronic ratio > 10).

### 4.2. Chronic Toxicity of Meloxicam

By contrast, chronic toxicity refers to the adverse effects of repeated or prolonged exposure to low doses over weeks or months. Such exposure often results in sublethal effects and may encompass a substantial portion of the organism’s life cycle (≥10%). Growth retardation, behavioural abnormalities, and reproductive malfunction are examples of such outcomes. Establishing emission regulations and creating environmental safety plans need an evaluation of such hazards [110].

In the carp *Cyprinus carpio*, prolonged exposure (21–28 days) to sublethal concentrations of meloxicam (0.1–2 mg/L) causes oxidative stress. This is indicated by a reduction in the activity of antioxidant enzymes (superoxide dismutase, catalase, and glutathione peroxidase) and the expression of corresponding mRNAs (up to 50%) in the gills, liver, kidneys, and brain, as well as an accumulation of malondialdehyde, the primary indicator of oxidative stress. Erythrocyte, haemoglobin, haematocrit, and alanine aminotransferase/alkaline phosphatase levels all rise. Furthermore, necrosis and hepatocyte vacuolization are seen at ≥1 mg/L, suggesting metabolic abnormalities [102].

Meloxicam exposure (0.1–1000 μg/L) for 31 days during the early stages of ontogenesis in freshwater fish *Oryzias melastigma* resulted in abnormalities in the development of the circulatory system and disturbance of digestive functions. Alongside these effects, there was a reduction in the expression of the *pla2* gene and malfunction of the renin-angiotensin system, which led to a buildup of angiotensin II [111].

Meloxicam’s specific inhibition of COX-2 appears to be connected to the mechanism of its toxicity. Na+/K+-ATPase activity in the gills decreases and oedema develops when prostaglandins, which are essential for renal perfusion and osmoregulation in fish, are suppressed. The damage is made worse by secondary oxidative stress cascades. Reactive oxygen species-mediated lipid peroxidation, DNA adduct production, and hepatocyte death are all influenced by glutathione and glutathione peroxidase deficiency [15].

Overall, information on the acute and chronic toxicity of meloxicam to non-target organisms remains very limited and is concentrated mainly on a single fish species. Data on invertebrates, plants, and fungi are scarce, which prevents a comprehensive environmental risk assessment. Although meloxicam is considered ‘vulture-safe’ relative to some other NSAIDs, its release into the environment may still pose broader ecosystem risks [112]. Controlling pharmaceutical active compounds requires restricting the use of meloxicam and giving eco-pharmacovigilance first priority. Future research should incorporate mixed effects and multigenerational analysis.

Thus, there are grounds to believe that the current risk assessment may underestimate some aspects of the impact of meloxicam, considering the fragmentary nature of the data and the lack of long-term studies. Available ecotoxicological evidence for meloxicam remains taxonomically narrow and is concentrated mainly on fish and a limited set of alternative models, which prevents a comprehensive environmental risk assessment and limits the extrapolation of current toxicity data to natural ecosystems.

## 5. (Bio)removal of Meloxicam

The removal of pharmaceutical active compounds from wastewater is essential to reduce long-term environmental risks. However, pharmaceutical residues are often only partially removed, typically by less than 50% [84,101,113,114]. Advanced physicochemical and biological strategies may improve degradation efficiency, reduce toxicity, and support the development of sustainable treatment technologies [101,114]. The mechanics, efficacy, and pertinent uses of both novel and current meloxicam detoxification techniques are discussed in this section.

### 5.1. Physicochemical Treatment of Meloxicam

According to recent research, meloxicam is effectively degraded by physicochemical methods employing nanomaterials, oxidative processes, and ultrasonication. By breaking down the molecule’s stable thiazole and benzothiazine fragments, these techniques offer a viable substitute for conventional pharmaceutical wastewater treatment techniques. These approaches can achieve removal rates of 70–100% under controlled laboratory conditions (pH, catalyst concentration, and energy consumption), but their scalability is limited by high energy demand, the formation of by-products and the need to assess the toxicity of intermediate and final products [115,116].

Photocatalysis with TiO_2_ nanoparticles under UV light (1012 µW/cm^2^) guarantees the breakdown of 77.34% of meloxicam (64.57 µg/mL) in 8 h (at pH 9.0 and a catalyst concentration of 0.4 mg/mL). The aromatic ring is attacked by hydroxyl-xylyl radicals to initiate the reaction [117].

Meloxicam degrades by 50–100% depending on the circumstances after 45 days of exposure to sunshine in model and natural river water, producing seven transition products. The free-living ciliate infusoria *Tetrahymena pyriformis* was toxically affected by one of the metabolites [74].

Heterostructured catalysts have been suggested as a solution to the drawbacks of pure semiconductors (charge recombination, limited absorption range). Because of the synergy between adsorption and the photo-Fenton process, which involves active forms ·OH, h+, e−, and O_2_^−^, the photocatalyst based on the heterojunction S-scheme FeWO_4_/ZnIn_2_S_4_ removes 98.7% of meloxicam (50 mg/L) and 92.9% of tetracycline (60 mg/L) [118]. The Z-scheme uses a similar interfacial charge transfer principle: the solid-state heterojunction FeCo-LDH/Ag/MIL-88B with Ag nanoparticles guarantees 99.3% meloxicam elimination because of the increased formation of O_2_^−^, ·OH, and h+, which induces cycle opening [119].

Unlike photocatalysis, which requires activation by irradiation, adsorption-based approaches enable the concentration of the contaminant without external energy input. Magnetic Fe_3_O_4_ composites encapsulated within the metal–organic framework MIL-100 (specific surface area > 2000 m^2^/g) remove meloxicam and naproxen via π–π interactions and electrostatic forces, thereby facilitating both material separation and reusability [120].

Ultrasonic activation is a useful substitute for photocatalysis when medium turbidity prevents it from being used. 75.7% of meloxicam (10 mg/L) will be broken down by sonocatalytic degradation utilising hydrothermally synthesised ZnWO_4_ in 120 min at an ultrasonic power of 0.278 W/cm^2^. Alongside the process, ·OH and h+ are produced, which causes C-N bonds to break and CO_2_ and inorganic compounds to form [115].

The employment of synthetic catalysts unites all the options outlined (Table 3). Enzymatic degradation, which imitates biological oxidative processes, provides a radically different strategy. In the presence of H_2_O_2_, artificial myoglobin-based peroxidase guarantees full meloxicam (50 µM) degradation in 90 s with a catalytic efficiency of kcat/Km 15.7 mM^−1^s^−1^. The thiazole ring is oxidised and then degraded into low-molecular-weight compounds, according to UPLC-MS data [121].

Nevertheless, the high efficiency of physicochemical methods is often achieved at the cost of increased capital and operational expenses. Such technologies are usually formed at the expense of electricity, reagents, electrode or membrane replacement, and handling secondary streams [65,122,123]. Life cycle assessments confirm the same logic. The removal of pharmaceutical active compounds reduces the toxicological load, but energy-dependent post-treatment units can substantially increase the system’s own environmental load [124]. However, their economic viability depends on the type of matrix, the required depth of mineralisation, the cost of energy, and the need for subsequent treatment of effluents, sludge, or oxidation products [125,126].

Despite the high efficiency (up to almost 100% removal of meloxicam) in laboratory conditions, the application of such technologies is limited by energy costs, the formation of by-products, and the need for subsequent assessment of the toxicity of the decomposition products of the compounds.

### 5.2. Microorganism-Based Bioaugmentation of Meloxicam

Bioaugmentation relies on the enzymatic capabilities of microorganisms that can degrade xenobiotics through co-metabolism or as the sole carbon source. It is attractive because of its selectivity, low energy demand, absence of secondary contamination, and adaptability to real-world matrices [127].

Despite these benefits, there are still a lot of unanswered questions about the biodegradation of meloxicam. Specifically, there is a dearth of information on this compound’s introduction, quantitative evaluation, and ecological behaviour.

Eukaryotic microorganisms are the subject of the oldest data. Meloxicam (2 mg/L) was reportedly converted by the filamentous zygomycete *Cunninghamella blakesleeana* NCIM 687 by 75.72% over the course of five days, resulting in three by-products, including two important mammalian metabolites, 5′-hydroxymethylmeloxicam and 5′-carboxymeloxicam [128,129].

Bacterial meloxicam degraders are not as thoroughly researched as fungi, and their efficiency is much lower.

Enterobacteria (*Enterobacter aerogenes*, *Escherichia coli*), firmicutes (*Bacillus cereus*, *B. subtilis*), and pseudomonads (*Pseudomonas aeruginosa*, *P. putida*) were found to biodegrade meloxicam under optimal conditions. Biodegradation was most pronounced in *B. subtilis* MTCC 441 (27.17%) and *P. putida* NCIM 2782 (41.15%), with 5′-hydroxymethylmeloxicam as the main metabolite [130].

In a recent study, we showed that meloxicam can be bioaugmented by actinomycetes of the genus *Gordonia* from the Regional Specialised Collection of Alkanotrophic Microorganisms (acronym IEGM, the World Federation for Culture Collections # 285, http://www.iegmcol.ru, accessed on 26 May 2026), producing similar products. We discovered that the strain *Gordonia alkanivorans* IEGM 1277 could totally break down 10 mg/L of meloxicam in 14 days [75]. Phenotypic, metabolic, and ultrastructural alterations in bacterial cells have been linked to adaptive reactions of *Gordonia* to the effects of meloxicam.

There is a lot more information available on bioaugmentation of other NSAIDs. Using a variety of bacterial species, including *Pseudomonas*, *Bacillus*, *Rhodococcus*, and others [131], significant data on the bacterial breakdown of diclofenac [132], ibuprofen [133], and naproxen [134], as well as their combinations [135,136,137,138], have been gathered.

The available literature suggests that meloxicam exhibits limited susceptibility to bioaugmentation (Table 4).

Available data indicate that meloxicam undergoes bioaugmentation rather than proven complete mineralisation in most studied systems. Fungal strains, particularly *Cunninghamella blakesleeana*, show higher conversion rates than the bacterial isolates reported so far, whereas bacterial degradation has been demonstrated only for a limited number of taxa under laboratory conditions. The identified products are mainly hydroxylated and carboxylated derivatives, but the fate of downstream intermediates and the extent of total carbon conversion remain insufficiently resolved.

The financial outlook for biological methods appears more favourable compared to the application of physicochemical methods. Bioaugmentation using microorganisms is usually considered a cost-effective and less energy-intensive option, especially when the goal is to reduce organic load and detoxify pharmaceutical pollutants without expensive post-treatment [139,140]. However, savings are often accompanied by incomplete removal of resistant compounds, long hydraulic retention times, and the risk of residual toxicity in the treated water or sludge [126]. Moreover, bioaugmentation may incur costs for membranes, aeration, and maintaining the operational parameters of the system [140]. In summary, physicochemical methods often excel in the depth and speed of xenobiotic removal but fall short in terms of energy, reagents, and secondary impacts, whereas biological methods are usually cheaper and more environmentally friendly in operation but weaker against the most resistant compounds.

Overall, the data on bioaugmentation with microorganisms is based on the study, as a rule, of a single strain under controlled conditions. Therefore, the transferability of the results to natural communities and large-scale purification processes requires further research. However, bioaugmentation should be considered a promising option for the detoxification of meloxicam and related micropollutants in water and soil, particularly in comparison with energy-intensive physicochemical methods. Its main advantages are low energy demand, environmental compatibility, and the possibility of application in mixed-contaminant systems. Nevertheless, further work is needed to improve process predictability, broaden strain diversity, and verify performance under field conditions.

## 6. Meloxicam and Other Oxicams: Environmental Assessment

Other oxicams, such as piroxicam, is also detected in wastewater, surface waters, and sediments in trace levels [74,141]. For several oxicams (e.g., lornoxicam and tenoxicam), data are largely limited to analytical or pharmacological studies. There are direct measurements in hospital wastewater in Iran, where average concentration of piroxicam was 6.32 ± 2.5 µg/L [142]. Review studies highlight piroxicam, meloxicam, and tenoxicam as environmentally significant enolic NSAIDs that are poorly removed by standard wastewater treatment plants and therefore enter aquatic ecosystems [15].

Photodegradation appears to be the major natural pathway for oxicams transformation. Tenoxicam, piroxicam, and meloxicam (2 µg/L) in river water with partially limited sunlight exposure persisted for 15, 27, and 45 days, respectively. Meloxicam therefore showed higher persistence [74]. This may be related to the acid–base properties of meloxicam. The pKa of meloxicam is 4.2, whereas that of tenoxicam and piroxicam is around 5.2, which affects ionisation, solubility, and transport in the medium.

From a practical standpoint, high-energy electron beam treatment of piroxicam shows a high degree of decomposition at 98.98% in model water and 89.6% in real wastewater [142]. However, such approaches are associated with significant financial costs. By contrast, natural transformation rates are low, so even trace concentrations continue to pose environmental risk.

In summary, the data suggest that oxicams present an ecologically relevant group of NSAIDs and may persist in aquatic ecosystems even at low concentrations. In this context, meloxicam appears to warrant particular attention due to its potential environmental impact. The available evidence indicates that it may be relatively more resistant to natural degradation. This in turn supports further investigation of its environmental safety and effective removal methods.

## 7. Conclusions

Pharmaceutical environmental contamination is a complex and constantly changing problem that needs both international legal control and a scientific response. Meloxicam is considered an underestimated pharmaceutical contaminant, with its occurrence in aquatic ecosystems and isolated cases of ecotoxicity in fish being most reliably documented. Biodegradation remains the most promising strategy. However, this requires a broader range of studied microorganisms, analysing metabolites, and validation of real applicability in treatment systems.

In conclusion, we note a substantial lack of published data on the occurrences and behaviour of meloxicam in soils, flora, and fauna, which limits robust assessments of its ecological risk. Further research is necessary to fill this gap. We believe that it is necessary to focus on the following priority areas: (1) targeted monitoring programmes using validated analytical methods (LC-MS/MS, GC-MS) for soil, plant biomass, animal tissues, and animal products (milk, meat, eggs, and related matrices); (2) the development of standardised sampling protocols; (3) laboratory studies on microbial resistance and bioaugmentation; and (4) ecotoxicological and bioaccumulation studies (tests on algae, invertebrates, and fish) to assess toxicity and the potential for biomagnification. These measures would help in the targeted identification of meloxicam in environmental matrices and, over time, reduce uncertainties in assessing its ecological risk.

## Figures and Tables

**Table 1:** The main areas of application of meloxicam in human and veterinary medicine.

Category	Direction for Use, mg	Ref.
Adults, third molar extraction	PO, 7.5	[25]
Adults, tooth impaction (acute pain)	IV, 15–60	[26]
Various surgeries (orthopaedics, abdominal, colorectal)	IV, 30	[27,28,29]
Cats, osteoarthritis (chronic pain)	PO, 0.01–0.1 (per kg of weight)	[30]
Dogs, ovariohysterectomy	IV, 0.2 (per kg of weight)	[31]
Dogs, TPLO (knee surgery)	SC, 0.1–0.2 (per kg of weight)	[32]
Dogs, oestrus (fertility)	SC, 0.2 (per kg of weight)	[33]

PO—*per os*, IV—*intravenous*, SC—*subcutaneous*.

**Table 2:** Solubility, lipophilicity, and polymorphism of meloxicam.

Parameter	Characteristic	Ref.
IUPAC name	4-Hydroxy-2-methyl-*N*-(5-methyl-2-thiazolyl)-2*H*-1,2-benzothiazine-3-carboxamide-1,1-dioxide	[39]
Molecular formula	C_14_H_13_N_3_O_4_S_2_	[39,40]
CAS number	71125-38-7	
Molecular weight, g/mol	351.40	
Boiling point, °C	581.30	
Melting point, °C	254.00	
pKa	1.1 and 4.2	[36]
Log K_ow_	3.43	[36,41]
Prototype forms	Anion, acidic enol, zwitterion, and cation; the form depends on pH and solvent; in neutral and slightly alkaline environments, the anionic form predominates.	

**Table 3:** Comparative performance and limitations of meloxicam removal strategies.

Method	Typical Conditions/Principle	Reported Effect on Meloxicam, %	Main Limitations	Ref.
Photocatalysis on TiO_2_	UV-activated oxidation; pH 9.0; catalyst 0.4 mg/mL; 8 h	77.34	Requires UV irradiation; catalyst recovery may be needed; only laboratory-scale evidence	[117]
Natural degradation	Sunlight exposure in model and natural river water; 45 days	50–100	Slow process; toxicity of metabolites must be assessed	[74]
S-scheme FeWO_4_/ZnIn_2_S_4_	Adsorption/photo-Fenton process with ROS generation	98.7	Needs engineered nanomaterial and external energy input; scalability uncertain	[118]
Z-scheme FeCo-LDH/Ag/MIL-88B	Adsorption–photodegradation with enhanced ROS formation	99.3	Complex synthesis; cost and regeneration issues	[119]
Core–shell Fe_3_O_4_ @MIL-100(Fe)	Adsorption via π–π electrostatic interactions	40–80	Does not mineralise the compound; sorbent regeneration required; capacity depends on pH and competing solutes	[120]
Sonocatalytic degradation with ZnWO_4_	Ultrasound-activated degradation; 120 min	75.7	Energy-intensive; efficiency depends on turbidity and reactor design	[115]
Artificial peroxidase based on myoglobin scaffold	Biomimetic oxidation in the presence of H_2_O_2_; 90 s	100	Enzyme stability, cost, and peroxide management limit practical use	[121]

**Table 4:** Comparative performance and limitations of meloxicam biodegradation.

Method	Typical Conditions/Principle	Reported Effect on Meloxicam Decomposition, %	Main Limitations	Ref.
Fungal bioaugmentation by *Cunninghamella blakesleeana*	Biotransformation over 5 days	75.72	Biotransformation rather than full mineralisation; only few strains studied	[129]
Bioaugmentation by selected bacterial strains	*Enterobacter*, *Bacillus*, *Pseudomonas* under optimal conditions	27.17–41.15	Low efficiency; data fragmented; conditions not directly comparable across studies	[130]
bioaugmentation by *Gordonia alkanivorans* IEGM 1277	Biodegradation of 10 mg/L meloxicam in 14 days	Complete breakdown of parent compound	Complete mineralisation not demonstrated; performance shown for a single strain under controlled conditions	[75]

## Data Availability

No new data were created or analysed in this study. Data sharing is not applicable to this article.
